# A Species Conservation-Based Particle Swarm Optimization with Local Search for Dynamic Optimization Problems

**DOI:** 10.1155/2020/2815802

**Published:** 2020-08-01

**Authors:** Dingcai Shen, Bei Qian, Min Wang

**Affiliations:** ^1^College of Mathematics and Computer Science, Gannan Normal University, Ganzhou 341000, China; ^2^School of Computer and Information Science, Hubei Engineer University, Xiaogan 432000, China; ^3^Finance Office, Gannan Normal University, Ganzhou 341000, China

## Abstract

In the optimization of problems in dynamic environments, algorithms need to not only find the global optimal solutions in a specific environment but also to continuously track the moving optimal solutions over dynamic environments. To address this requirement, a species conservation-based particle swarm optimization (PSO), combined with a spatial neighbourhood best searching technique, is proposed. This algorithm employs a species conservation technique to save the found optima distributed in the search space, and these saved optima either transferred into the new population or replaced by the better individual within a certain distance in the subsequent evolution. The particles in the population are attracted by its history best and the optimal solution nearby based on the Euclidean distance other than the index-based. An experimental study is conducted based on the moving peaks benchmark to verify the performance of the proposed algorithm in comparison with several state-of-the-art algorithms widely used in dynamic optimization problems. The experimental results show the effectiveness and efficiency of the proposed algorithm for tracking the moving optima in dynamic environments.

## 1. Introduction

As a very challenging optimization tool, evolutionary algorithms (EAs) have been successfully applied to the optimization problems in static environments. Nevertheless, EAs have not been effectively used to solve optimization problems in dynamic environments, which are very common in many real-world applications, for example, the changes of vehicle routing due to the temporary traffic control or sudden changes in weather, the newly added artefacts in production scheduling, and uncertain market factors lead to changes in financial trading models. In these complex real-world problems, its constraints and coefficients or even objectives may vary with time. The problems with these characters can be modelled as dynamic optimization problems (DOPs). In DOPs, the worse candidate solutions in the past can be the optimal solutions in the new environment, and vice versa. The development of solution strategies in the area of EAs that may work in such uncertain environments raises new challenges.

In static environments, the goal is to find a single optimum or multioptima in the search space. However, in dynamic environments, the goal of the algorithms is no longer to locate the optimal solution, but to continuously track the moving optimum as closely as possible. Many researchers have introduced various strategies into canonical EAs to enhance their ability for tracking changing optima. In static optimization, convergence is a positive factor for the algorithms to locate the global optimum; however, it may weaken the ability of the algorithm to find the moving optimum in a later evolving process because of the diversity loss. In order to increase or maintain the population diversity, researchers have developed many schemes to enhance the canonical EAs' ability to locate moving optimum in dynamic environments.

The simplest method of solving DOPs is to regard each change as the new optimization and reinitialize the population. However, in real-world applications, most of the environmental changes may not be too drastic, and the new optimum will be in some sense related to the historical optimal solutions [1]. In that case, saving the old optimum according to certain strategies is beneficial to the optimization in the new environment. Thus, the memory-based scheme is a widely used method adopted in DOPs [2–4]. In [2], the authors combined the multipopulation scheme and an improved memory strategy, which is saving the elite individual retrieved from each subpopulation, to enhance the exploration ability of the algorithm. In [4], a similar technique as [2] is used for the optimization of DOPs.

A similar scheme to memory named species is also a commonly used technique by EAs' community [5]. Species-based EAs are often regarded as a kind of multiswarm algorithm. It preserves the candidate solutions distributed in the search space according to a predefined radius, and after the evolution in each generation, the saved species seeds are either replacing the worse individual, if there has an unprocessed individual within the search radius, or replacing the worst unprocessed individual in the current population, if there is no solution within the search radius [6]. Many experimental results have shown that multiswarm strategy is helpful in locating multiple optima in multimodal optimization problems, and some researchers have extended this strategy to DOPs and achieved a good performance in locating and tracking the moving optimum [7, 8].

In EAs' community, there are two well-known strategies, which are also commonly adopted in the optimization of DOPs to promoting diversity of the population, named hypermutation [9] and random immigrants. Hypermutation increases population diversity by drastically increasing the mutation rate as the environment changes, and random immigrants strategy replaces a fraction of EAs' population at each generation. The selection of individuals to be replaced often has two ways: one is randomly selected to be replaced and this way seems to be losing the good candidates in some cases, and another one is replacing the worst individuals in the current population.

Particle swarm optimization (PSO) [10] is a population-based stochastic optimization algorithm; it can be considered as one of the most popular nature-inspired metaheuristics for continuous optimization, which was first developed by Eberhart and Kennedy in 1995. Due to its simple concept and easy implementation, PSO has been developed rapidly in the last two decades [11]. PSO has been widely used in the optimization in static environments, and many important research studies have been achieved [12–14]. In recent years, with more and more attention being paid to the research area of DOPs, PSO has been widely applied to the optimization of DOPs.

According to the characteristics of DOPs, and the commonly adopted strategies introduced mentioned above, we observed that the population diversity and historical information play an important role in the optimization of DOPs. Motivated by these observations, a species conservation combined with a spatial neighbourhood best searching strategy is integrated into canonical PSO (denoted as sslPSO) during the evolutionary process, to strengthen the exploration and exploitation ability of the PSO. In each iteration, in order to mitigate the loss of population diversity, the best individual in each subswarm is archived, and then, these species seeds are transferred into the next generation. Experiments on a commonly used benchmark of moving peaks benchmark (MPB) are carried out to investigate the effect of our proposed algorithm. The experimental results show that the proposed algorithm has a promising performance in solving the DOPs.

The primary contribution of sslPSO is an enhanced species conservation, which uses species conserving during the environmental change period; at the same time, the spatial neighbourhood searching is introduced in the updating procedure. The comparison results indicate that the introduction of species conservation is beneficial for the tracking of moving optima in dynamic environments. Meanwhile, the spatially local best searching in the updating of position ensures the algorithm's high exploration ability while maintaining high exploitation ability.

The rest of this paper is arranged as follows. In Section 2, the canonical PSO and its application in DOPs and some related works included in this study are briefly reviewed. The proposed sslPSO algorithm is described in detail in Section 3.  Section 4 compares the sslPSO with other state-of-the-art algorithms widely used in DOPs which are presented to show the effectiveness and efficiency of the proposed algorithm. Finally, Section 5 concludes the study and outlines future work.

## 2. Background and Related Works

In this section, we first give a brief description of the definition of DOP. After that, the PSO framework and its application in DOPs and the need of species in dynamic environments are given briefly.

### 2.1. DOP

Dynamic optimization problems are optimization problems in which one or more of its problem parameters, such as objective function, constraints, or environmental coefficients, may change over time. The dynamic character of the problem makes the optimization procedure much more complicated than the static optimization problem.

A DOP can be defined in general as follows:

(1) $$\begin{array}{c} \left\{\begin{array}{cc} \text{opt} & f\left(x,t\right),\\ \text{s}.\text{t}. & g_{i}\left(x,t\right)\leq 0,\;\left(i=1,2,\ldots ,m\right),\\ \; & h_{j}\left(x,t\right)=0,\;\left(j=1,2,\ldots ,n\right), \end{array}\right. \end{array}$$

where *f*(**x**, *t*)is the objective function of the problem. **x** is the decision vector of *D*dimensions in the search space, and *g*_*i*_(**x**, *t*) and *h*_*j*_(**x**, *t*) denote the *i*th inequality constraint and *j*th equality constraint of the problem, respectively. Both of them may vary during the evolutionary process for specific change type in real-world applications. In our study, we only consider the dynamic optimization without any dynamic constraints.

### 2.2. Particle Swarm Optimization (PSO)

In a PSO algorithm, each particle *i* is a potential solution in the *D*-dimensional search space. The position of *i*th particle in a population with *N* individuals (**X**_1_, **X**_2_,…**X**_*N*_) can be represented by **X**_*i*_=(*X*_*i*1_, *X*_*i*2_,…, *X*_*i* *D*_), and the velocity of this particle is represented by **V**_*i*_=(*V*_*i*1_, *V*_*i*2_,…, *V*_*iD*_). In a commonly used update model, each particle is updated by two best solutions, **P**_*i*_=(*P*_*i*1_, *P*_*i*2_,…, *P*_*i* *D*_), the best position of particle *i* it has experienced, and **P**_*g*_=(*P*_*g*1_, *P*_*g*2_,…, *P*_*gD*_), the best position discovered so far by all particles. At the beginning, *N* particles are randomly generated over the search space. Then, all particles search for the optimum by fly over the search space, until the global optimal position is found. At each iteration, the new velocity and position of each particle can be updated according to its current velocity and position as follows [10]:

(2) $$\begin{array}{c} V_{i}\left(t+1\right)=\omega V_{i}\left(t\right)+\varphi_{1}\left(P_{i}\left(t\right)-X_{i}\left(t\right)\right)+\varphi_{2}\left(V_{g}\left(t\right)-X_{i}\left(t\right)\right), \end{array}$$

(3) $$\begin{array}{c} X_{i}\left(t+1\right)=X_{i}\left(t\right)+V_{i}\left(t+1\right), \end{array}$$

where

(4) $$\begin{array}{c} \varphi_{1}=c_{1}R_{1},\\ \varphi_{2}=c_{2}R_{2}, \end{array}$$

*ω* is a parameter used to control the influence degree of the previous velocity to the current velocity, which is named inertia weight. *c*_1_ and *c*_2_ are two positive constants balancing the contribution of the particle's own previous best position and the best position the whole swarm had attained. **R**_1_ and **R**_2_ are two *D*-dimensional random vectors uniformly distributed in the interval *U*(0.0, 1.0).

In the velocity updating procedure using equation (2), all particles are attracted to the best solution (*P*_*g*_(*t*)) found by all members of the population. This updating model is typically called global best (denoted as gbest). In the gbest model, every individual learns from its own experience and imitates the very best member found in the population [15]. Another commonly used model is called local best (denoted as lbest). In the lbest network, each individual's movement is attracted by the best performance of its neighbours in a certain range. The determination of neighbours usually has two ways: one is determined according to the index which is randomly assigned at the initialization step and keeps constant in the whole evolution process; the other is determined on the basis of spatial distance between particles (the difference between these two structures will be described in detail in Section 3). The gbest model is generally considered to have a faster convergence rate; in the meantime, it would be more likely to get stuck in local optimal solution. The lbest model, however, is converged slower with less likely to be trapped into the local optimum than that of gbest model. The PSO algorithm repeatedly applies the update procedure until the maximum evaluations are reached. The pseudocode of the canonical PSO algorithm is described in Algorithm 1.

### 2.3. PSO in Dynamic Optimization Problems

The application of PSO to dynamic optimization problems has been widely studied in recent years [5, 16–20]. Similar to other EAs, there are two key facts that must be faced in the application of PSO to dynamic environment: one is the outdated memory, and the other is the loss of diversity [18]. Most of the existing research studies introduce various strategies into canonical PSO to overcome these two problems. When a new environment comes, the previous best location visited by the particle becomes outdated. In this case, a simple and effective response is to recalculating the fitness value of the objective function of each particle. However, in real-world applications, most of the environmental changes are usually not dramatic. When the dynamic problem changes periodically or recurrently, it might be helpful to reuse previously found solutions to reduce the computation times [21]. Many researchers adopt various strategies to save redundant information to the memory particles for later use in case of environmental changes. In [22], a triggered memory scheme is proposed, in which the best individuals found by the “explore” population are stored in the memory, and two memory retrieval schemes, named memory-based resetting and memory-based immigrants, are adopted to retrieve memory when environmental change is detected. Zhu et al. [4] propose a new memory scheme with a large memory capacity to improve the performance of multipopulation algorithms for DOPs.

In dynamic optimization, convergence is unfavourable to the performance of the algorithms. Therefore, many researchers have introduced various schemes into canonical PSO to maintain or increase the diversity of the population during evolution. Inspired by the movement of atom, Blackwell et al. [23, 24] proposed charged PSOs to maintaining diversity in the solving of DOPs. Multiple swarm/population methods are often considered as another effective strategy for increasing population diversity in the optimization of DOPs. In [25], the authors proposed two multiswarm paradigms of particle swarms and verified the effectiveness on a widely used moving peaks benchmark. Li et al. [8] proposed an adaptive multiswarm optimizer to enable adaptively to change the number of populations in dynamic environments.

### 2.4. The Need of Species Conservation

In biology, species are a group of creatures with similar characteristics; similarly, in EAs' community, a species represents a collection of individuals with common characteristics. In all similarity measurements, the Euclidean distance is the most commonly used similarity measurement. The smaller the distance between two individuals, the more similar they are.

The distance between two individuals **X**_*i*_=(*X*_*i*1_, *X*_*i*2_,…, *X*_*iD*_) and **X**_*j*_=(*X*_*j*1_, *X*_*j*2_,…, *X*_*jD*_) is defined as follows:

(5) $$\begin{array}{c} d\left(X_{i},X_{j}\right)=\sqrt{\sum_{k=1}^{D}{\left(X_{i,k}-X_{j,k}\right)}^{2}}. \end{array}$$

In this paper, the above definition of distance is used to measure the similarity between two individuals.

**Figure 1:**
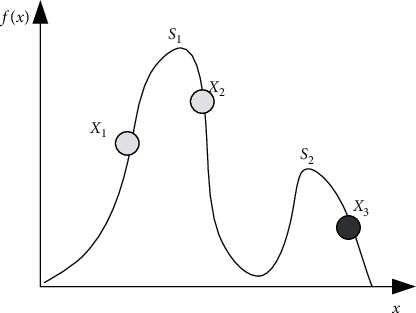
An illustration of the need for species conservation.

Figure 1 illustrates the need for species conservation during evolution. As Figure 1 shows, after some iterations of the EA, most of the individuals have converged on peak *S*_1_, and at the same time, an individual *X*_3_ with low fitness has emerged. Because of its low fitness, the individual *X*_3_ will have a high probability of being eliminated in the next generation. However, this individual is very important in the multimodal optimization problems that need the algorithms to find all the optima, or in dynamic optimization problems that need the algorithms to track the moving optima. In order for the individual to survive in the next generation, this individual must be preserved. The species conservation procedure is described in Algorithm 2.

## 3. The Proposed Algorithm

Our proposed algorithm, denoted by sslPSO, takes advantage of the memory strategy to enhance the tracking ability of the PSO in dynamic environments. After initialization, the proposed enhanced species conservation-based PSO algorithm conducts the main loop with four main stages: species determination, particle update, seeds conservation, and particle attractor and swarm attractor update. These stages are briefly described as follows.

The species-based scheme is introduced into the multimodal optimization or dynamic optimization [17], in which the whole population is divided into several species according to their similarity. In the later of the evolution process, most individuals are concentrated in a few optima, resulting in too large number of individuals in the corresponding species and a rapid decline in population diversity. Thus, a mechanism for preventing too many individuals following a single peak is needed for the algorithm to track the moving optima. As in [17], a parameter *PS*_max_ is also introduced in our study. If the number of particles in a species exceeds *PS*_max_, only the pre-*PS*_max_ fittest particles are retained in the species, and the remainder will be randomly reinitialized, thus to maintain the population diversity during the evolution.

Following the species determination, the update procedure is applied. In this study, an improved lbest-based update strategy is introduced. In the usual lbest topology, each particle is affected by the best performance of its *r* immediate neighbours. However, the vector indices are assigned as their generating order at the initialization step and are kept constant throughout the evolution. The indices-based neighbours may belong to a different group far apart spatially in the search space, resulting in a degradation of the exploitation ability of the algorithm.

**Figure 2:**
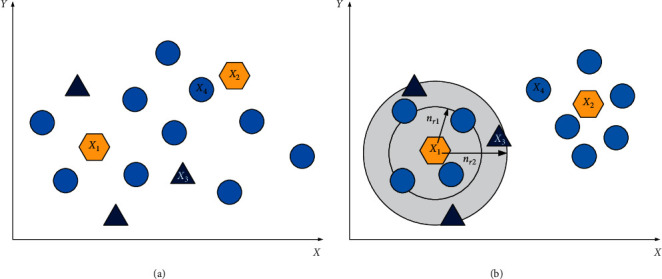
The illustration of different neighbourhood structures: (a) indices-based neighbourhood and (b) spatial-based neighbourhood.

As Figure 2(a) shows, the individuals **X**_1_, **X**_2_, **X**_3_, and **X**_4_ are neighbours to each other, but it can be seen from the figure that some of them are spatially far apart. Take **X**_1_ as an example, if an index-based neighbour is used, then **X**_2_ or **X**_3_ will be selected as the local best particle, and **X**_1_ searches around **X**_2_ or **X**_3_, resulting in severe decrease in the exploitation ability of the algorithm. Different from the indices-based neighbourhood, we use spatial-based neighbourhood in the update procedure, and the parameter *r* is renamed as *neighbourhood radius* and denoted by *n*_*r*_, which is used to search the fittest individual in what range. The determination of local best in *n*_*r*_ neighbours is as shown in Algorithm 3. From Figure 2(b), we can see that the update of **X**_1_ is only affected by the individuals around it in a certain range. If the radius is small, i.e., *n*_*r*1_, then the update of **X**_1_ is affected by 4 individuals (denoted by the solid blue circle); if the radius is further increased, i.e., (*n*_*r*1_ < *n*_*r*2_), then the update of the **X**_1_ will be affected by 7 individuals around it. The difference in neighbourhood radius will affect the individual's exploitation ability and exploration ability. If the neighbourhood radius is small, the algorithm's exploitation ability will be higher; otherwise, the algorithm's exploration ability will increase. In dynamic environments, how to achieve a balance between exploitation ability and exploration ability is an important fact for the algorithm to improve its performance. The performance of the algorithm under different neighbourhood radius will be analysed later in this paper.

In order to accelerate the convergence process over an environment period, the inertia weight *ω* in equation (2) is linearly decreasing from *ω*_max_ to *ω*_min_ as follows:

(6) $$\begin{array}{c} \omega =\omega_{\text{max}}-\frac{\left(\omega_{\text{max}}-\omega_{\text{min}}\right)\times E_{\text{itr}}}{E_{\text{itr}}}, \end{array}$$

where *ω*_max_ and *ω*_min_ are the maximum and minimum values of *ω*, respectively, and *E*_itr_ is the iteration counter during an environment cycle (in this study, it is assumed that the environmental change cycle is known in advance).

Once all the particles are updated, some species may not survive. Thus, seeds conservation process is conducted immediately after the update procedure. The species seeds are either copied to the new population or substituted by better samples of the same species.

**Figure 3:**
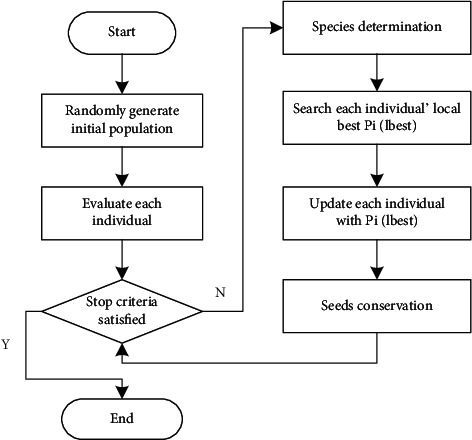
Flowchart of sslPSO.

Algorithm 4 describes the framework of the sslPSO and Figure 3 presents the flowchart of the algorithm.

## 4. Experimental Evaluation

In this section, a series experiments are carried out based on the moving peaks benchmark problem to measure the effectiveness of the proposed algorithm (run on an Intel Core i5 4590@ 3.30 GHz processor with 8 Gb RAM on Windows 10 Home Premium 64-bit operation system), and then, the performance of sslPSO is compared with a set of EAs taken from the literature for DOPs. The involved algorithms include CPSO [19], mCPSO [24], mQSO [24], SPSO [26], and rSPSO [27] and canonical PSO. All the results of the peer algorithms presented in this paper are taken from the paper where they were proposed. In the following section, we will introduce the benchmark function adopted in our experiments, as well as the performance measure and parameter settings. Finally, we present the experimental results and analysis.

### 4.1. Benchmark Problem

The moving peaks benchmark (MPB) was proposed by Branke [28] and has been widely used to test the performance of the dynamic optimization algorithms. Within an MPB problem with *N*_*p*_ peaks in a *D*-dimensional search space, the location, height, and the width of the peaks can be varied at a certain frequency. The form of the canonical MPB is formulated as follows:

(7) $$\begin{array}{c} F\left(X,t\right)=\underset{i=1,\ldots ,N_{p}}{\max}\frac{H_{i}\left(t\right)}{1+W_{i}\left(t\right)\sum_{d=1}^{D}{\left(X_{d}\left(t\right)-X_{id}\left(t\right)\right)}^{2}}, \end{array}$$

where *W*_*i*_(*t*) and *H*_*i*_(*t*) denote the width and height of peak *i* at time *t*, respectively, and *X*_*id*_(*t*) denotes the *d*th element of peak *i* at time *t*, and *X*_*d*_(*t*) is the *d*th element of particle **X** at time *t*.

The location of each peak is shifted by a vector *v* of a fixed distance *s* in a random direction. The parameter *s* is named as the shift length, which is used to define the change severity of the dynamic problems. The move of a single peak can be defined as follows:

(8) $$\begin{array}{c} v_{i}\left(t\right)=\frac{s}{\left|r+v_{i}\left(t-1\right)\right|}\left(\left(1-\lambda \right)r+\lambda v_{i}\left(t-1\right)\right), \end{array}$$

where **r** is a random vector and *λ* is used to determine the move direction correlated to the previous movement, *λ*=0 for a random direction and *λ* > 0 for a direction related to the previous direction.

More formally, a move of a single peak can be denoted as follows:

(9) $$\begin{array}{c} H_{i}\left(t\right)=H_{i}\left(t-1\right)+{\text{height}}_{\text{severity}}\times \sigma , \end{array}$$

(10) $$\begin{array}{c} W_{i}\left(t\right)=W_{i}\left(t-1\right)+{\text{width}}_{\text{severity}}\times \sigma , \end{array}$$

(11) $$\begin{array}{c} X_{i}\left(t\right)=X_{i}\left(t-1\right)+v_{i}\left(t\right), \end{array}$$

(12) $$\begin{array}{c} \sigma \in N\left(0,1\right). \end{array}$$

### 4.2. Performance Measurement

Properly measuring the performance of the algorithms is vital in the optimization of DOPs. There are several most commonly used criteria to evaluate the algorithms in existing studies. Existing performance measures in DOPs can be classified into two main groups: optimality-based and behaviour-based. Interested readers can refer to [21] for a more detailed description. In this study, in order to quantify the performance of the proposed algorithm (sslPSO) within a dynamic environment, and for a fair comparison with the peer algorithms, we used the MPB as the test suite, and the offline error measurement is adopted, which is defined as follows:

(13) $$\begin{array}{c} e_{\text{off}}=\frac{1}{N_{e}}\sum_{k=1}^{N_{e}}\left(f_{k}^{\text{opt}}-f_{k}\right), \end{array}$$

where *f*_*k*_ is the best evaluation found by an algorithm right before the *k*th environmental change, *f*_*k*_^opt^ is the theoretical optimum value of the *k*th environment, *e*_off_ is the average of all differences between *f*_*k*_^opt^ and *f*_*k*_ over the environmental changes, and *N*_*e*_ is the total number of environmental changes in a run.

### 4.3. Parameter Settings

The default settings of the MPB used in the experiments in this paper are given in Table 1, which are the same as in all the peer algorithms (for SPSO in [26], the authors in [27] tuned the algorithms to optimising this benchmark, and the results are taken from [27]). In Table 1, the term “change of frequency, *U*” means that for every *U* fitness evaluation, an environment change will occur. Initially, *P* peaks are randomly generated with the given boundaries of position, height, and width as shown in Table 1. The height and width are shifted randomly with the shift severity *s* in the range *H*=[30,70] and *W*=[1,12], respectively.

In our sslPSO algorithm, the population size is set to 100, and the learning factors *c*_1_ and *c*_2_ are both set to 1.7. The inertia weight *ω* is initially set to *ω*_max_=0.9 and then decreases linearly to *ω*_min_=0.3 over the entire change cycle. The other parameters are set as follows: the species size is confined to PS_max_=10, the species distance *σ*_*s*_ is set to 30, the *neighbourhood radius* is set to *n*_*r*_ = 3.0, and the performance with different sizes is also investigated in our study. For each test case, there were *N*_*e*_  = 100 environmental changes, which result in *N*_*e*_ × *U* = 100 × 5,000 fitness evaluations in each run. The result of each experiment is the average of 30 independent runs with different random seeds.

### 4.4. Experimental Results and Analysis

In this section, the performance of sslPSO is investigated in several aspects, including the effect of neighbourhood radius, the effect of varying the shift severity, and the ability of locating and tracking the moving optima, respectively.

#### 4.4.1. Sensitivity Analysis of Parameter *n*_*r*_

**Figure 4:**
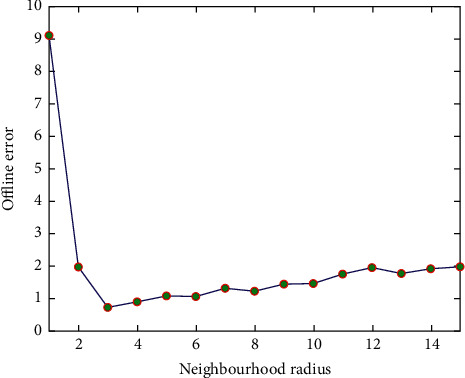
Offline error of sslPSO on the MPB problem with different neighbourhood radius.

Figure 4 presents the offline error achieved by sslPSO on the MPB problem with different neighbourhood radius. From Figure 4, we can see that when the particles learn from only its nearest neighbour (i.e., the neighbourhood radius is set to 1.0), the sslPSO achieved the largest offline error. With the increase of neighbourhood radius, the offline error begins to decrease. When the neighbourhood radius reaches 3.0, the optimal solution obtained is 0.75 and then increases slowly with the increase of the neighbourhood radius. When the neighbourhood radius is equal to the popsize, the lbest model is equivalent to gbest model, and the offline error obtained by the algorithm is 6.56 (not plotted in Figure 4).

As can be seen from Figure 4, when the neighbourhood radius is small, the algorithm has a good balance of exploration ability and exploitation ability. As the neighbourhood radius increases, the individual moves closer to the global optima at a faster rate, which weakens the exploration ability of the algorithm. And this is the key issue that needs to be overcome in the optimization problem in the dynamic environments.

#### 4.4.2. Comparison of sslPSO with Peer Algorithms

In this section, a set of experiments is conducted to compare the performance of sslPSO with peer algorithms on the MPB problems with different settings of the shift severity parameter*s*.  Table 2 presents the experimental results regarding the offline error and standard deviation. The experimental results of the peer algorithms are taken from the corresponding research studies, and the parameters are set to the optimal values which enable them to achieve their best performance (e.g., using the optimal configuration of C(70, 3) for CPSO and using the optimal *n*_*r*_=3.0 for sslPSO).

From Table 2, it can be seen that the results achieved by sslPSO are much better than the results of the other five algorithms on the MPB problems with different shift severities and are better than the results of the CPSO in most cases, or at least as good as CPSO. As we have speculated, the performance obtained by the canonical PSO is the worst among all algorithms, that is, the canonical PSO almost loses the ability to track moving optima in a dynamic environment. As we know, with the increasing of the shift severity, the ability of the algorithm to track the moving peaks decreases simultaneously. From the results of Table 2, we can see that the performance of all the algorithms degrades when the shift severity increases (there is an exception that canonical PSO achieves the similar worst performance under different shift severities). The offline error of SPSO increases fastest in comparison with the other five algorithms, while the sslPSO and CPSO are two algorithms which are slightly affected by the increase of the shift severity. When the shift severity is set to 0.0, i.e., there is no movement of the peaks, the offline error of the sslPSO achieved the best with 0.65. When the shift severity is set to 1.0, a value setting as most researches adopted, the sslPSO achieved a value of 0.75, which is much better than the other peer algorithms. When the shift severity reaches 6.0, a value which is usually hard for an algorithm to track the moving optima, the offline error of sslPSO is 1.92, which is only slightly higher than that of CPSO, and is much better than the results of the other five algorithms. The results show that sslPSO is very robust to track the moving optima in severely change environments.

## 5. Conclusions

Particle swarm optimization algorithms have been widely used in the optimization in static environments, and some promising results have been achieved in recent years when it was applied to address DOPs. For DOPs, in order to effectively track the moving optima in dynamic environments, it is usually important to introduce additional strategies to increase or maintain the population diversity or to effectively use the history optima information in the following evolution.

In this work, a species conservation-based PSO combined with a spatial neighbourhood best searching is proposed for DOPs. In order to effectively track the moving optima in dynamic environments, the previously found optima distributed in the population are reserved according to their dissimilarity based on Euclidean distance and are either preserved or replaced by the better individuals within a predefined range in the following evolution. Experimental results on a commonly used benchmark function for DOPs show that the proposed algorithm can greatly improve the performance of PSO in terms of tracking the moving optima in a dynamic fitness landscape with multiple changing peaks. The performance of sslPSO has good expansibility regarding the change severity in the peaks movement in comparison with other peer algorithms. sslPSO performs much better than mCPSO, mQSO, rSPSO, SPSO, and PSO in tracking the moving optima in dynamic environments with different change severities and is better than CPSO or as good as it in each circumstance. When the change severity is small, sslPSO outperforms all the other peer algorithms. In future work, we will consider using fewer memory optima to reduce the computational complexity caused by the participation of the previous optima in the subsequent evolution; it would also be very interesting to investigate the performance of the proposed technique under different change periods and change peaks, and applying to the real-world application is also a promising direction.

## Figures and Tables

**Algorithm 1:**
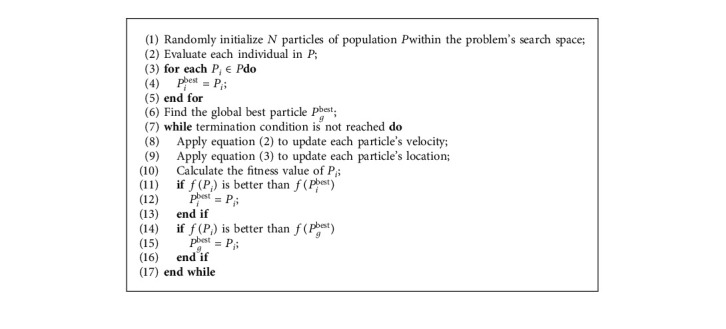
Pseudocode of the canonical PSO algorithm.

**Algorithm 2:**
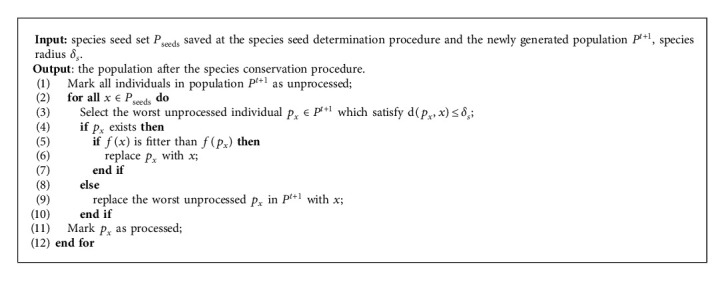
The procedure of species conservation.

**Algorithm 3:**
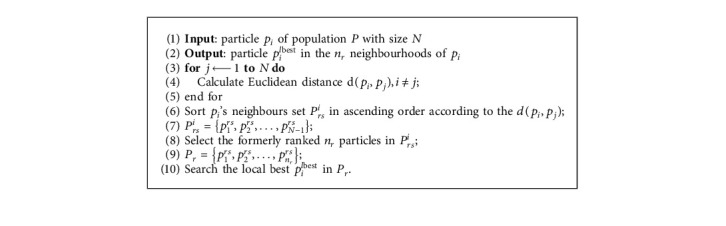
The algorithm of spatially neighbourhoods best searching.

**Algorithm 4:**
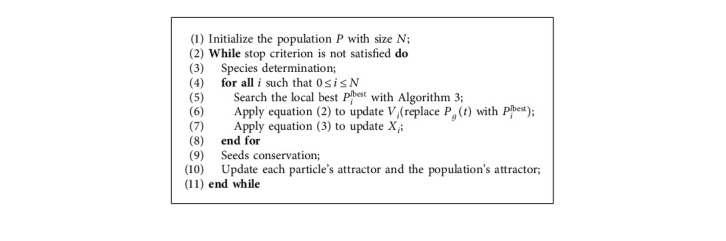
The framework of sslPSO.

**Table 1:** Default settings for the MPB problem.

Parameter	Value
Number of peaks, *P*	10
Change of frequency, *U*	5000
Height severity	7.0
Width severity	1.0
Peak shape	Cone
Shift length, *s*	1.0
Number of dimensions, *D*	5
Search space range	[0, 100]
Peak height, *H*	[30, 70]
Peak width, *W*	[1, 12]
Correlation coefficient, *λ*	0

**Table 2:** Offline error of algorithms on the MPB problems with different shift severities.

*s*	sslPSO	CPSO	mCPSO	mQSO	rSPSO	SPSO	PSO
0.0	0.65	0.80	1.18	1.18	0.74	0.95	15.47
	±0.22	±0.21	± 0.08	±0.08	±0.08	±0.09	±2.29
1.0	0.75	1.06	2.05	1.75	1.50	2.51	16.75
	±0.25	±0.24	±0.07	±0.06	±0.08	±0.09	±2.89
2.0	1.18	1.17	2.80	2.40	1.87	3.78	14.91
	±0.28	±0.22	±0.07	±0.06	±0.05	±0.09	±3.24
3.0	1.32	1.36	3.57	3.00	2.40	4.96	15.45
	±0.24	±0.28	±0.08	±0.06	±0.08	±0.12	±3.32
4.0	1.59	1.38	4.18	3.59	2.90	2.56	15.42
	±0.22	±0.29	±0.09	±0.10	±0.08	±0.13	±3.42
5.0	1.59	1.58	4.89	4.24	3.25	6.76	16.36
	±0.24	±0.32	±0.11	±0.10	±0.09	±0.15	±4.23
6.0	1.92	1.53	5.53	4.79	3.86	7.68	15.35
	±0.22	±0.29	±0.13	±0.10	±0.11	±0.16	±4.52

## Data Availability

No data were used to support this study.
